# Optimized Paocai Fermentation of Rehmannia Radix: Physicochemical Quality, Untargeted Metabolomics, and Antioxidant Activity

**DOI:** 10.3390/foods15173097

**Published:** 2026-08-31

**Authors:** Jilun Hu, Xiaoming Huang, Jing Xie, Xiaoyun Hou, Yingjie Li, Yunfei Zheng, Juan Li, Lianzhen Li, Erwei Li, Liya Hong

**Affiliations:** 1 College of Agronomy, Henan Agricultural University, Zhengzhou 450046, China; 17839340918@163.com (J.H.);; 2 United Graduate School of Chinese Modern Agriculture, Zhengzhou 450046, China; 3 Institutional Center for Shared Technologies and Facilities, Institute of Microbiology, Chinese Academy of Sciences, Beijing 100101, China

**Keywords:** Rehmannia Radix, paocai, fermentation, response surface methodology, electronic tongue, untargeted metabolomics

## Abstract

Rehmannia Radix is an important medicinal and edible plant resource in China. However, the production of paocai by fermenting Rehmannia Radix has rarely been reported. This study employed response surface methodology to optimize fermentation conditions, and investigated the changes in physicochemical properties, bioactive components, and metabolic profiles of paocai before and after fermentation. The optimal fermentation conditions were 5% salt, 9% sugar, and fermentation at 20 °C for 5 days. During the fermentation process, total acidity increased, pH decreased, and crispness and chewiness declined significantly, indicating a softer texture. Electronic tongue analysis demonstrated that fermentation decreased bitterness and astringency, while enhancing sourness, sweetness, and umami. Fermentation significantly increased total phenolic content, total flavonoid content, and antioxidant activity, with phenolic content increasing from 0.80 to 1.63 mg/g and flavonoid content increasing from 2.67 to 3.67 mg/g, while the contents of iridoid glycosides and phenylethanoid glycosides decreased significantly after fermentation. Untargeted metabolomics analysis identified 719 metabolites, of which 417 were differentially abundant metabolites, indicating that fermentation altered the metabolic profile of paocai. In conclusion, fermentation exerted a bidirectional regulatory effect: mitigating the bitterness and astringency of Rehmannia Radix paocai while improving its texture and flavor. This study not only developed the optimal fermentation process for Rehmannia Radix-based medicinal and edible products, but also provided the scientific basis for the sustainable processing and high-value utilization of Rehmannia Radix as a functional food.

## 1. Introduction

Rehmannia Radix (RR) is used medicinally as fresh, raw, or processed tuberous roots of *Rehmannia glutinosa* Libosch. in China, which is a perennial herb in the Scrophulariaceae family [1]. RR is regarded as one of the four principal Huai medicinal herbs, which are predominantly cultivated in some regions such as Henan, Shanxi and Shandong provinces in China [2]. The primary chemical constituents of RR, including iridoids, phenylethanoid glycosides, and saccharides, exhibit significant pharmacological activities, including hypoglycemic, anti-inflammatory, and antioxidant effects [3]. Fresh Rehmannia Radix (FRR) is famous for its ability to clear heat and cool the blood, and is traditionally used to treat conditions such as heat syndrome, sore throat, constipation, and hematemesis [4,5]. RR has long been used for both medicinal and edible purposes, and its earliest record dating back to Shennong’s Classic of Materia Medica. In 2024, RR was officially listed in the Catalog of Medicine-Food Homology of China. Medicine-food homologous materials refer to substances that possess both the nutritional value of food and the therapeutic effects of herbal medicines, thereby providing both nutritional and health benefits [6]. Previous research has mainly focused on pharmacological mechanisms, processing technologies, and clinical medicinal applications. After RR was added to the catalog of medicinal and edible homologous materials, research on it faces new challenges, including optimizing food processing, reducing astringent taste, improving solubility, and efficiently retaining active ingredients. These emerging research directions may greatly enrich the industrial development potential and theoretical research system of RR resources. Overall, RR boasts broad industrial and research prospects for future development.

Paocai is a traditional fermented food originating from China, Korea, and other East Asian countries, which is widely consumed as a side dish or appetizer. Common raw materials include radish, mustard greens, long beans, among others, which are fermented into paocai through either spontaneous or inoculated fermentation. These products are highly favored by consumers for their distinctive flavors and rich nutritional profiles [7,8,9]. Fermentation is a traditional processing technique that transforms complex compounds into more biologically active forms. In recent years, it has gained increasing popularity in food processing [10]. This processing method significantly enhances the flavor and nutritional profiles of functional foods [11] and provides various health benefits, including antioxidant [12], anti-inflammatory [13], anti-obesity [14], and cholesterol-lowering effects [15]. Recent studies have shown that fermentation greatly improved the aroma profile of plant-based protein extracts [16]. Khan et al. reported that fermentation decreased the levels of bitter amino acids in dried longan, and simultaneously improved its antioxidant activity [17]. The fermentation of sea buckthorn fruit transformed malic acid into lactic acid and carbon dioxide, which enhanced its flavor profile [18]. Furthermore, recent research has shown that the fermentation of radish paocai imparted floral, sweet, and sour flavors, thereby enhancing its overall flavor profile [19]. Chung et al. demonstrated that fermenting *Panax ginseng* Meyer with *Lactococcus lactis* improved the contents of total phenolics and flavonoids, as well as the antioxidant activity [20]. Qiao et al. demonstrated that fermentation significantly promoted the accumulation of polysaccharides, saponins, and flavonoids in *Astragalus membranaceus* [21]. Based on these findings, fermentation has gradually become a research hotspot in the field of functional food processing.

Based on our lab’s preliminary experiments, we found that fermenting FRR effectively reduced its bitterness and greatly improved the sensory quality and overall acceptability. Fermentation enabled room-temperature preservation of FRR with low salt, reduced post-harvest loss of Huai Rehmannia Radix raw materials, and extended the deep processing industrial chain of medicinal and edible homologous resources. To improve the quality and fermentation efficiency of FRR paocai and promote its industrial production, this study focused on optimizing the fermentation process parameters of FRR paocai through response surface methodology. The multidimensional analytical framework was established to evaluate the impact of fermentation on the quality and bioactivity of FRR paocai. Electronic tongue technology was used to monitor the overall flavor characteristics of FRR before and after fermentation, and the contents of total flavonoids, total phenolics, as well as the antioxidant activity were measured to evaluate the variation patterns of functional components during fermentation. Untargeted metabolomics was employed to analyze the changes in the overall metabolic profile of FRR before and after fermentation, and differential metabolites were screened with the criteria of *p* < 0.05, fold change ≥2 or ≤0.5, and VIP ≥1. Finally, HPLC was employed to quantify the key bioactive compounds in FRR before and after fermentation. This study comprehensively analyzed the effects of fermentation on the quality and functional properties of FRR paocai from multiple aspects, including sensory evaluation, electronic tongue analysis, metabolite composition, and active ingredients. These findings may provide a feasible strategy for the value-added utilization of FRR resources and offer innovative perspectives and experimental evidence for the development of functional fermented foods derived from medicinal and edible homologous materials.

## 2. Materials and Methods

### 2.1. Chemical Reagents and Materials

Fresh Rehmannia Radix was harvested in December 2025 from a planting base in Wuzhi, Henan Province, China. It was authenticated as the ‘Jinjiu’ cultivar by Dr. Liya Hong of Henan Agricultural University and stored at −80 °C and 4 °C, respectively, for subsequent experiments. Acetonitrile, methanol, and formic acid, UPLC/MS grade, were provided by Thermo Fisher Scientific. Folin–Ciocalteu reagent, DPPH, ABTS, and other chemicals, as well as the standards including rehmannioside D, aucubin, melittoside, ajugol, salidroside, catalpol, and acteoside (all with purity >98%), were purchased from Shanghai Yuanye Bio-Technology Co., Ltd. (Shanghai, China).

### 2.2. Fermentation Process Optimization

#### 2.2.1. Fermentation Process

The fermentation method for FRR was established by our research group. Briefly, a total of 10 kg of FRR with a fresh, firm texture and an average individual root weight of approximately 150 g was selected as the raw material to ensure sample uniformity. After thorough rinsing, the FRR was cut into uniform strips (1 cm × 3 cm). To reduce bitterness and suppress microbial proliferation, the strips were soaked in 7% (*w*/*v*) saline solution for 6 h, then washed with distilled water. It should be noted that the salt used in this debittering step was not counted in the total salt addition during fermentation. Subsequently, the cleaned FRR strips were mixed with varying amounts of salt and sugar, along with 0.1% (*w*/*w*) paocai fermentation starter powder (Beijing Chuanxiu Co., Ltd., Beijing, China). The starter powder comprised *Lactobacillus plantarum*, *Lactobacillus acidophilus*, *Lactobacillus rhamnosus*, and maltodextrin as the carrier. The mixture was then transferred into sterilized 1.5 L pickling jars with 400 g of FRR each, and a 2 cm headspace reserved below the lid. The jars were sealed and fermented at a constant temperature of 20 ± 1 °C. Unfermented and fermented samples were immediately frozen at −80 °C for subsequent analysis and designated as R-NF and R-F, respectively.

#### 2.2.2. Response Surface Experiment

This experiment was performed to investigate the effects of fermentation time (1 d, 3 d, 5 d and 7 d), salt addition amount (3%, 4%, 5%, 6% and 7%) and sugar addition amount (8%, 9%, 10%, 11% and 12%) on FRR paocai. Sensory scores were measured separately under different fermentation conditions. The effects of different fermentation conditions on each of these indicators were analyzed and compared. Based on the single-factor experiment, the three factors and three levels were selected as follows: salt addition amount (A) (4%, 5%, 6%), sugar addition amount (B) (8%, 9%, 10%), fermentation time (C) (3 d, 5 d, 7 d). A Box–Behnken design comprising 17 experimental runs, including five replicates at the center point, was performed to optimize the fermentation parameters of FRR paocai using Design-Expert 13.0 software. Sensory evaluation scores served as the optimization indicator. The factor levels are shown in Table 1. Ten trained panelists (6 males, 4 females; aged 20−55 years), comprising both faculty members and students, were recruited from the College of Food Science and Technology, Henan Agricultural University, to conduct the sensory analysis. The specific sensory attributes evaluated are listed in Table S1. All samples were food-grade and prepared under strict hygienic conditions. Formal ethical approval was not required for this study. Verbal consent was obtained from all participants prior to their involvement in the study.

### 2.3. Physicochemical Analysis

The pH, total acidity, nitrite concentrations and texture of the sample were measured using the methods proposed by Zheng et al. and Ge et al. [22,23]. The pH value was determined with a pH meter (PHS−3E, INESA, Shanghai, China). Total acidity was measured by titration with 0.1 mol/L NaOH using phenolphthalein as an indicator, with a blank correction. The content (X, as lactic acid equivalents) was calculated as:

$$X\;=\;\frac{\left[c\;\times \;(V_{1}-V_{2})\right]\;\times \;0.090\;\times \;F}{m}\;\times \;1000$$

where: X: the total acid content (g/L); c: concentration of NaOH standard solution (mol/L); V_1_: titrant volume for sample (mL); V_2_: titrant volume for blank (mL); F: dilution factor; m: sample volume (mL).

Nitrite content was determined by the spectrophotometric method using sulfanilamide-N-(1-naphthyl)ethylenediamine at 538 nm. The samples were cut into 1 cm × 1 cm × 1 cm for texture profile analysis using a texture analyzer (Stable Micro Systems, Godalming, UK). The tests were performed using a P50 probe (Stable Micro Systems, Godalming, UK) with the following parameters: a test speed of 1 mm/s, a compression ratio of 50%, and an interval time of 5 s. The definitions of texture parameters were as follows: crispness (g): the peak force at the first major fracture peak during the first compression cycle. Cohesiveness: the ratio of the positive area under the second compression curve to that under the first compression curve. Gumminess (g): calculated as the product of hardness and cohesiveness. Springiness (%): the percentage of height recovery between the first and second compression cycles relative to the original compression distance. Chewiness (g): calculated as the product of gumminess and springiness.

### 2.4. Electronic Tongue Analysis

The tests were conducted using the methods proposed by Wang et al. and Liu et al. [24,25], with some minor modifications. A 20 g sample was mixed with 100 mL of distilled water. The mixture was homogenized, centrifuged, and the supernatant was collected for analysis. The electronic tongue taste analysis was conducted using the INSENT SA402B intelligent (INSENT, Atsugi, Japan) taste analysis system. This instrument was equipped with 9 sensors, which responded to sourness, bitterness, astringency, aftertaste-B, aftertaste-A, umami, richness, saltiness, and sweetness, respectively. Prior to each measurement, the sensors were subjected to a three-step cleaning procedure. In the first step, the sensors were immersed in an ethanol-containing cleaning solution for 90 s: the negatively charged membrane sensors were treated with an HCl solution containing 30% (*v*/*v*) ethanol, while the positively charged membrane sensors were treated with a KCl-KOH solution containing 30% (*v*/*v*) ethanol. In the second and third steps, the sensors were sequentially rinsed in the reference solution (30 mM KCl and 0.3 mM tartaric acid) for 120 s each. Taste values were obtained by recording the potential responses of the sensors (mV), which were generated through the interaction between the sensors and tastants. To ensure the reliability of the data and the validity of the statistical analysis, three replicates were set for each sample. PCA was performed using Python 3.11 Statistical significance of the differences in taste profiles among samples was determined by one-way ANOVA.

### 2.5. Analysis of the Total Content of Phenolics and Flavonoids

The R-F and R-NF samples were freeze-dried and ground into powder. They were mixed with 70% (*v*/*v*) aqueous methanol at a 1:10 ratio and ultrasonically extracted at 25 °C for 30 min. After centrifuging at 12,000× *g* and 4 °C for 15 min, the supernatant was used for subsequent analysis. The approach outlined was employed to assess the total phenolic content (TPC), with minor modifications. Specifically, the TPC in the samples was assessed via the Folin–Ciocalteu method. An aliquot of 0.1 mL of the sample methanol extract was combined with 0.5 mL of Folin–Ciocalteu reagent and allowed to react in the dark for 6 min. Subsequently, 0.8 mL of a 7.5% Na_2_CO_3_ solution was added to the mixture. The absorbance was recorded at 765 nm with a spectrophotometer (CLARIO star Plus, BMG LABTECH, Ortenberg, Germany) [26,27]. Gallic acid regression equation: *y* = 7.5073*x* + 0.0842, R^2^ = 0.9999. Results were expressed as milligrams of gallic acid equivalents per gram of dry weight.

According to Peng’s method, the total flavonoid content (TFC) in the samples was measured using the Al (NO_3_)_3_ colorimetric assay [28], with slight modifications. 0.2 mL of sample methanol extract was combined with 0.2 mL of a 5% NaNO_2_ solution and incubated for 6 min. After adding 0.2 mL of 10% Al (NO_3_)_3_ solution, the mixture was allowed to react for a further 6 min. Then, 2 mL of 4% NaOH was added, the mixture was shaken, and allowed to stand for 15 min. Absorbance at 510 nm was recorded using a spectrophotometer, as outlined by Wu et al. [29]. Rutin regression equation: *y* = 0.5413*x* + 0.0337, R^2^ = 0.9990. Results were expressed as milligrams of rutin equivalents per gram of dry weight.

### 2.6. HPLC Analysis of Bioactive Compounds

The contents of seven active components in R-F and R-NF, including the iridoid glycosides (catalpol, aucubin, rehmannioside D, melittoside, ajugol) and the phenylethanoid glycosides (salidroside, acteoside), were determined by an HPLC system. The samples were lyophilized, subsequently extracted with 25% (*v*/*v*) methanol under ultrasonication for 30 min, and centrifuged at 12,000× *g* and 4 °C for 10 min. The supernatant was filtered through a 0.22 µm membrane filter prior to HPLC analysis. Quantitative results of bioactive compounds were expressed as mg/g dry weight. Chromatographic separation was achieved on an HB C18 column (250 mm × 4.6 mm, 5 µm) (TUP Labs, Tianjin, China) maintained at 25 °C. Water (eluent A) and acetonitrile (eluent B) comprised the mobile phase, which was delivered at a rate of 1.0 mL/min. The injection volume was 10 µL. For the analysis of iridoid glycosides, the elution gradient: 0 min, 2% B; 8.0 min, 5% B; 22.0 min, 15% B. The detection wavelength was set at 203 nm. For the analysis of phenylethanoid glycosides, 0 min, 15% B; 18.0 min, 35% B; 25.0 min, 65% B. The detection wavelength was set at 275 nm and 334 nm. The column was equilibrated with the initial mobile phase for 5 min before each injection. Quantitative analysis was performed by constructing a standard curve using standard substances (Table 2). The results of the chromatographic analysis of the identified bioactive compounds were expressed as mg/g.

### 2.7. Untargeted Metabolomics Analysis by UPLC-Q-TOF-MS

R-F and R-NF samples were extracted with 70% (*v*/*v*) methanol-water solution at a ratio of 1:10 (*w*/*v*) by ultrasonication at 25 °C for 30 min. The extracts were then centrifuged at 12,000× *g* and 4 °C for 15 min, and the resulting supernatant was collected for analysis. Chromatographic column: ACQUITY UPLC BEH C18 (Waters, Milford, MA, USA) (1.7 µm × 2.1 mm × 100 mm); flow rate: 0.4 mL/min; injection volume was maintained at 2 µL; eluent A: 0.1% formic acid in water; eluent B: acetonitrile. The following multistep linear gradient was applied: 0 min, 2% B; 1.5 min, 2% B; 21.5 min, 100% B; 23.5 min, 100% B; 25 min, 2% B. Mass spectrometric detection was performed in negative ion mode using an electrospray ionization (ESI) source on a Xevo G2-XS QTOF (Waters, Milford, MA, USA). The ESI source was configured with the following settings: capillary voltage, 2 kV; acquired mass range (*m*/*z*), 100–1500 Da. The experiment included three independent biological replicates. Statistical significance was assigned to *p* < 0.05. Raw data were acquired using MassLynx V4.2 software. Progenesis QI 3.0 software was used to process the raw data, including peak identification, integration, retention time correction, peak alignment, and normalization. Signal intensities for metabolites below the limit of detection were recorded as zero. To further ensure data reliability, metabolic features with a missing rate exceeding 50% were subsequently excluded. Compound identification was performed using the Human Metabolome Database (HMDB) and METLIN database (2019).

### 2.8. Determination of the Antioxidant Activity

Accurately weighed sample powder (0.5 g) was extracted three times with 5 mL of 70% methanol under ultrasonication for 10 min each time. The combined extracts were centrifuged at 10,000× *g* for 10 min, and the supernatant was concentrated and freeze-dried. Subsequently, 50 mg of the lyophilized powder was weighed and reconstituted in 1 mL of methanol. The DPPH radical scavenging activity was assessed following the methodology outlined by Duan et al. and Wang et al. [30,31]. A total of 0.1 mL of each sample solution (0–6.0 mg/mL) was mixed with 0.1 mL of DPPH solution (0.2 mmol/L) and incubated in darkness for 30 min. The absorbance of the resulting mixtures was measured at 517 nm using a spectrophotometer (CLARIO star Plus, BMG LABTECH, Ortenberg, Germany). The ability to scavenge ABTS radicals was assessed according to previously described methods. 10 µL of each sample solution, ranging from 0 to 12.5 mg/mL in concentration, was mixed with 150 µL of ABTS working solution and incubated in the dark for 60 min. The absorbance values at 734 nm were recorded [32,33]. FRAP assay was performed following the method of Kaviya. Aliquots of 200 µL of R-F and R-NF samples (concentration range: 0–25 mg/mL) were mixed with 500 µL of phosphate buffer (0.2 mol/L, pH 7.4) and 500 µL of 1.0% potassium ferricyanide solution. The mixture was thoroughly vortexed and then incubated in a water bath at 50 °C for 20 min. After cooling to room temperature, 500 µL of 10% trichloroacetic acid solution was added, and the resulting mixture was centrifuged at 10,000× *g* for 10 min. The absorbance at 700 nm was measured [34,35]. The antioxidant activity of the samples was assessed by the DPPH and ABTS radical scavenging rates and the FRAP absorbance values. Higher scavenging rates and greater FRAP absorbance values indicated stronger antioxidant activity.

## 3. Results

### 3.1. Optimization of the Fermentation Process

#### Response Surface Optimization of FRR Paocai Process

**Figure 1:**
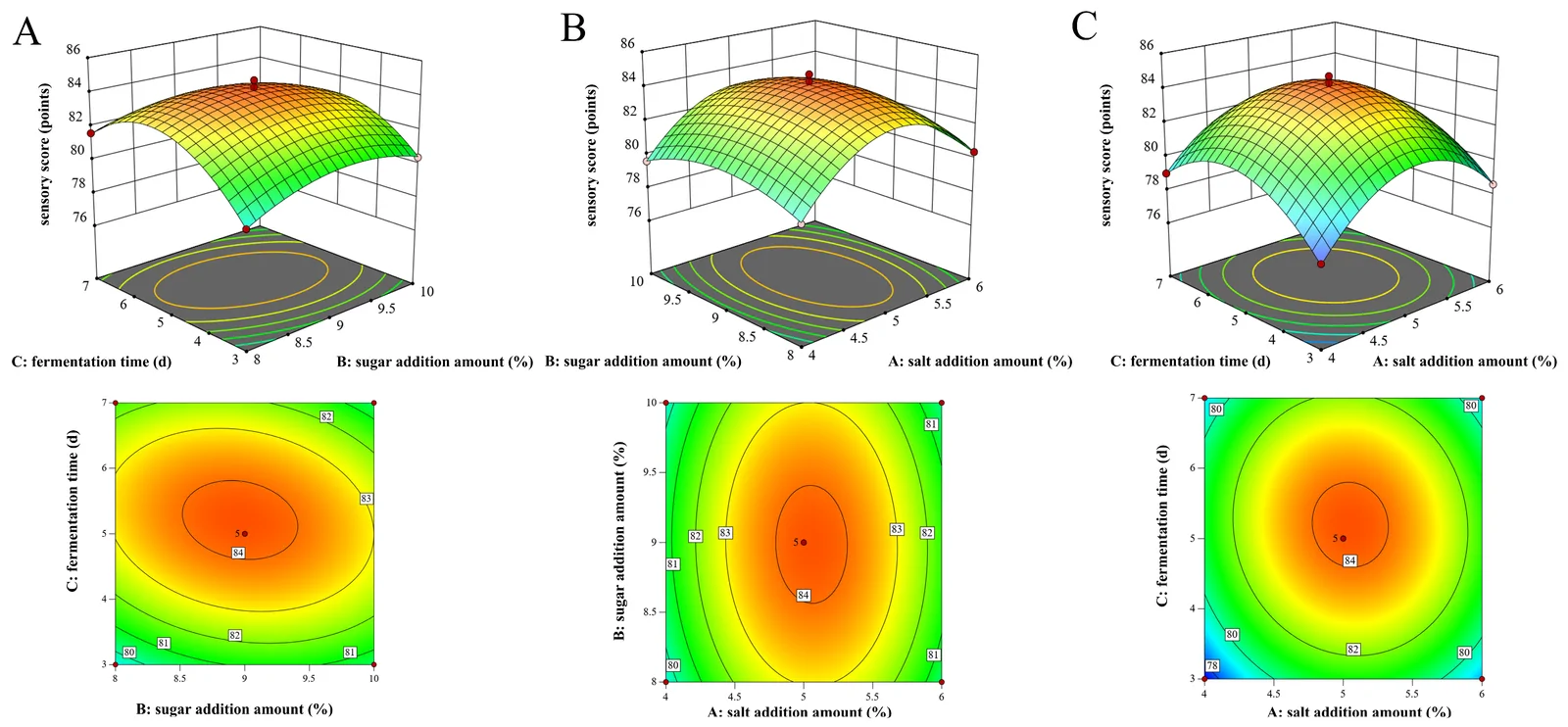
Response surface optimization results of FRR paocai: (**A**) Effect of fermentation time and the sugar addition amount. (**B**) Effect of the salt addition amount and the sugar addition amount. (**C**) Effect of the salt addition amount and fermentation time.

The optimization of fermentation conditions was based on single-factor experiments, with the sensory score (Y) adopted as the response value. The Box–Behnken response surface method was adopted to analyze the influence of each factor and the interaction effects among factors on the response value. The test results are shown in Supplementary Table S2, and the variance analysis results of this model are presented in Supplementary Table S3. The final fitting equation of FRR paocai was as follows: Sensory score (Y) = 84.20 + 0.3500 × A − 0.0250 × B + 0.5250 × C − 0.1000 × AB − 0.3000 × AC − 0.5500 × BC − 3.13 × A^2^ − 1.18 × B^2^ − 2.57 × C^2^. The value of *p* < 0.0001 indicated statistical significance and the missing fitting item was not significant, indicating that the model had a good fitting effect. The coefficient R^2^ of the model was 0.9924, and the prediction coefficient R_Adj_^2^ of the model was 0.9825, indicating that the model was feasible. By comparing the F values, it was observed that the influence of each factor on the sensory score of FRR paocai was as follows: fermentation time > salt addition amount > sugar addition amount. The response surface plot showed the influence of the interaction effects of various factors on the response value, as shown in Figure 1. Response surface analysis was used to optimize the production process of FRR paocai. The optimal conditions were determined to be a salt addition amount of 5.05%, sugar addition amount of 8.96%, and fermentation time of 5.21 days. Based on practical feasibility, the fermentation conditions were adjusted to 5% salt addition amount, 9% sugar addition amount, and a fermentation time of 5 days at 20 °C. Under these conditions, the sensory score of FRR paocai reached 84.80 ± 0.84 points.

### 3.2. Physicochemical Analysis

**Figure 2:**
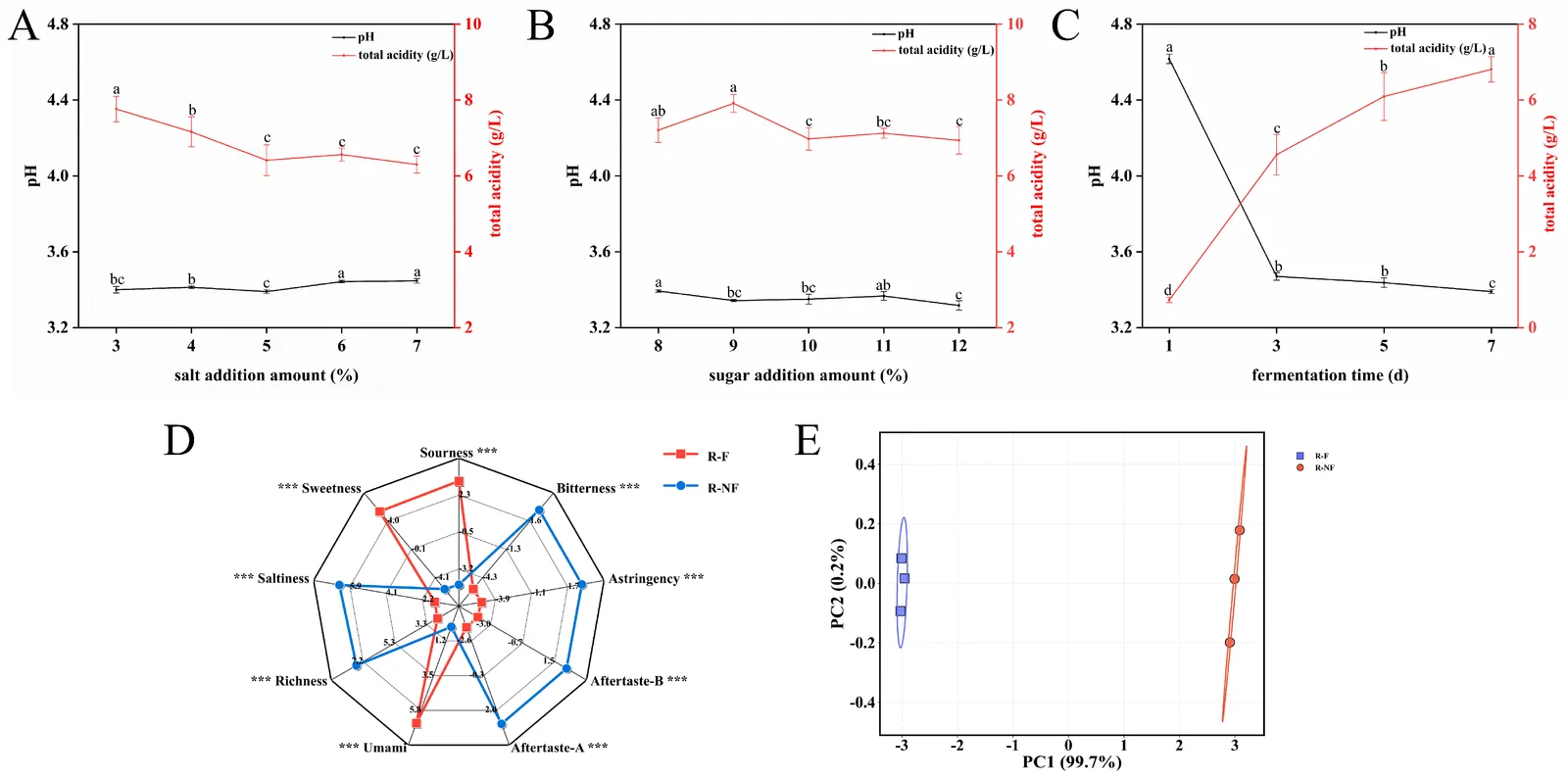
(**A**) The effects of different salt addition amounts, (**B**) sugar addition amount, (**C**) and fermentation time on the pH and total acid of paocai. Electronic tongue results of paocai before and after fermentation through radar map (**D**) and PCA (**E**). *** indicates a statistically significant difference at *p* < 0.001. Statistical differences at a 5% significance level were indicated by the superscript letters.

pH and total acidity are critical physicochemical parameters that determine the quality of paocai, as they directly reflect product quality and the dynamics of microbial activity during fermentation [36]. As shown in Figure 2A, the pH of FRR paocai exhibited an increasing trend with rising salt concentration, while the total acid content showed a decreasing trend. This phenomenon may be attributed to the fact that high salt concentrations inhibited the metabolic activity and reproduction of microbiota. According to Figure 2B, as the sugar concentration increased, the total acid content first increased and then decreased, peaking at a sugar concentration of 9%, whereas the pH generally demonstrated a declining trend. Sugar serves as an important carbon source for microbiota. An appropriate concentration of sugar promoted their growth and reproduction, leading to a significant advantage in the initial stage. However, excessive sugar concentration enhanced osmotic pressure, which adversely affected the growth of microbiota. During the entire fermentation process of FRR paocai, as the metabolic activities of microbiota occurred, the pH value gradually decreased while the total acid content gradually increased. The nitrite content in FRR paocai was less than 20 mg/kg, which complied with the Chinese National Standard GB 2762-2022 [37]. Recent studies have indicated that paocai is typically considered to have reached fermentation maturity and to be safe for consumption when the pH value is lower than 4.0 and the total acidity content is greater than 3 g/L [38,39]. In this study, by the third day of fermentation, FRR paocai had reached the fermentation maturity standard, with a pH of 3.47 and a total acidity of 4.56 g/L.

Texture reflects the changes in the softness, tissue structure and textural properties of paocai during fermentation and is an important indicator of the quality of paocai. The changes in crispness, springiness, chewiness, gumminess, and cohesiveness of FRR paocai during the fermentation process are shown in Table 3. The crispness, chewiness and gumminess of FRR paocai gradually decreased with fermentation, which is consistent with the results of previous reports. The reason may be that pectinase and cellulase produced by microbial activity decompose the tissue structure and cell wall of paocai, resulting in softening. The springiness of paocai generally exhibited a declining trend during the fermentation process. No significant differences in springiness were observed from days 1 to 7 of fermentation. However, the springiness values for all these time points were lower than that of the unfermented control. A reduction in cohesiveness suggests that the paocai could endure less destructive force. No significant differences in cohesiveness were observed during the first 3 days of fermentation; however, the cohesiveness of the paocai decreased after 5 days of fermentation. During fermentation, the textural properties of FRR paocai generally declined, with the texture softening while remaining within the range typical for paocai.

### 3.3. Electronic Tongue Analysis of FRR Before and After Fermentation

The results of the electronic tongue test are shown in Figure 2. The results revealed significant differences in taste between R-F and R-NF (*p* < 0.001). PCA explained 99.7% and 0.2% of the total variance by PC1 and PC2. The electronic tongue radar map indicated that R-NF showed higher response values in bitterness, astringency, aftertaste-B, aftertaste-A, richness, and saltiness, whereas R-F exhibited higher responses in sourness, umami, and sweetness. Fermentation significantly reduced bitterness, astringency, aftertaste-B, and aftertaste-A in FRR, while enhancing sourness, umami, and sweetness, thereby enriching the flavor profile of the paocai and imparting a palatable taste.

### 3.4. Total Phenolic and Total Flavonoid Content

Phenolic and flavonoid compounds, which are widely distributed in various plants as important secondary metabolites, exhibit significant biological activities. Research findings have demonstrated that fermentation can markedly increase the total phenolic and total flavonoid contents. The TPC of R-F (1.63 ± 0.02 mg/g) was significantly higher than that of R-NF (0.80 ± 0.01 mg/g) (*p* < 0.001). The TPC of R-F increased by 2.04-fold compared with that of R-NF. The TFC of R-F (3.67 ± 0.07 mg/g) was significantly higher than that of R-NF (2.67 ± 0.06 mg/g) (*p* < 0.001). The TFC of R-F increased by 1.37-fold compared with that of R-NF. Liu et al. indicated that fermentation enhanced the flavonoid content in dandelions compared to unfermented samples [40]. The increase in TPC and TFC was attributed to fermentation, which promoted the release of phenolic and flavonoid compounds and converted bound phenolics into free forms [19]. Fermentation facilitated the release of bioactive compounds from plants [41]. Meanwhile, microbial metabolic activities released phenolic and flavonoid compounds by degrading proteins, pectin, and other constituents [42].

### 3.5. Analysis of Bioactive Compounds in FRR Before and After Fermentation

As shown in Table 4, the contents of catalpol, aucubin, rehmannioside D, melittoside, ajugol, salidroside, and acteoside in R-NF were 45.77 ± 0.15 mg/g, 0.34 ± 0.01 mg/g, 2.51 ± 0.01 mg/g, 0.32 ± 0.03 mg/g, 2.45 ± 0.02 mg/g, 0.72 ± 0.00 mg/g, and 0.19 ± 0.01 mg/g, respectively, which are generally consistent with previous reports [43,44]. In contrast, the contents in R-F were 0.69 ± 0.08 mg/g, 0.02 ± 0.00 mg/g, 0.57 ± 0.01 mg/g, 0.07 ± 0.01 mg/g, 0.94 ± 0.00 mg/g, 0.52 ± 0.01 mg/g, and 0.08 ± 0.00 mg/g. The results indicated that fermentation led to a significant reduction in the contents of bioactive compounds in FRR (*p* < 0.05). This finding is consistent with the conclusion of Zhang et al. [45]. The reason may be that the acidic environment during fermentation promoted the hydrolysis of glycosides, and the β-glucosidase secreted by the metabolic activity of microbiota could hydrolyze the glycosidic bonds of glycoside compounds. Although the fermentation process reduced the content of glycosides, it may have generated new fermentation metabolites, giving FRR paocai a unique flavor and different biological activities.

### 3.6. Identification and Analysis of Metabolites

#### 3.6.1. The Composition of Metabolites

**Figure 3:**
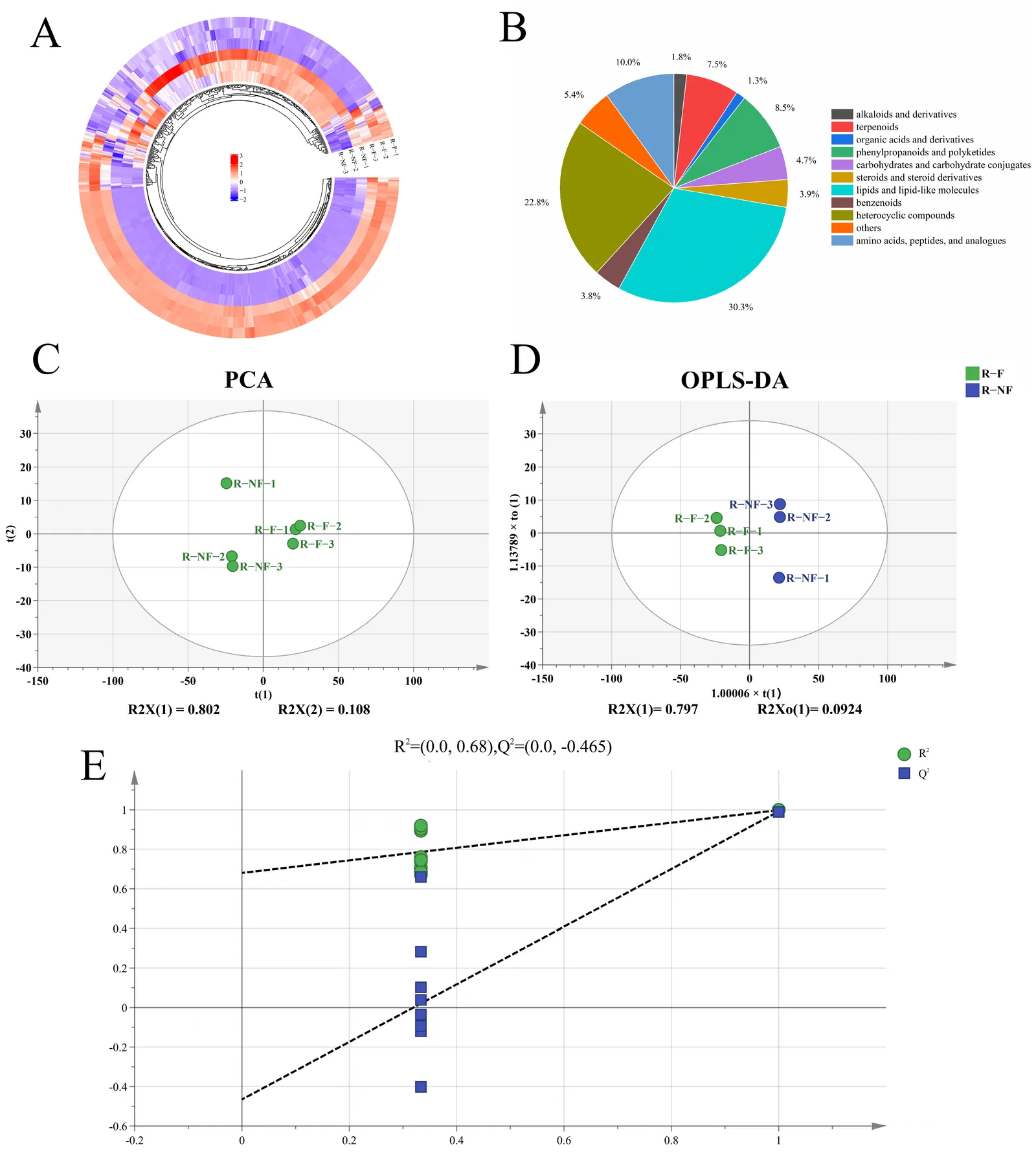
(**A**) HCA of R-F and R-NF. (**B**) The schematic representation of the different types of metabolites. (**C**) PCA score plot of R-F and R-NF. (**D**) OPLS-DA score plot of R-F and R-NF. (**E**) Permutation test results of the R-F group and R-NF group. R-F-1 to R-F-3: Biological triplicates of R-F samples; R-NF-1 to R-NF-3: Biological triplicates of R-NF samples.

The total ion chromatograms of R-F and R-NF are shown in Supplementary Figure S1. In this study, 719 metabolites were identified from R-F and R-NF in negative ion mode (Supplementary Table S4). As exhibited in Figure 3A, hierarchical cluster analysis (HCA) was performed on all metabolites. The HCA heatmap displayed the relative metabolite contents, revealing significant differences among the sample groups. All detected metabolites were categorized into 11 distinct classes. Figure 3B presents pie charts to offer a clearer understanding of the composition and classification of these metabolites. The main categories of metabolites were 13 alkaloids and derivatives (1.8%), 54 terpenoids (7.5%), 9 organic acids and derivatives (1.3%), 61 phenylpropanoids and polyketides (8.5%), 34 carbohydrates and carbohydrate conjugates (4.7%), 28 steroids and steroid derivatives (3.9%), 218 lipids and lipid-like molecules (30.3%), 27 benzenoids (3.8%), 164 heterocyclic compounds (22.8%), 72 amino acids, peptides, and analogues (10.0%), and 39 others (5.4%).

#### 3.6.2. Multivariate Statistical Analysis

To precisely evaluate the metabolic differences between R-F and R-NF, principal component analysis (PCA) was performed, and the results are presented in Figure 3C. Based on the PCA score plot for R-F and R-NF, PC1 contributed 80.2%, while PC2 contributed 10.8%. Significant differences were observed in the principal components between R-F and R-NF. The OPLS-DA score plot indicated significant distinctions between R-F and R-NF. The R^2^Y and Q^2^ values were 0.993 and 0.984, which were close to 1, indicating that the model was valid and reliable. During the modeling process, a random permutation test with 200 iterations was used to assess the suitability of the model and confirmed no overfitting.

#### 3.6.3. Analysis of Differential Metabolites

**Figure 4:**
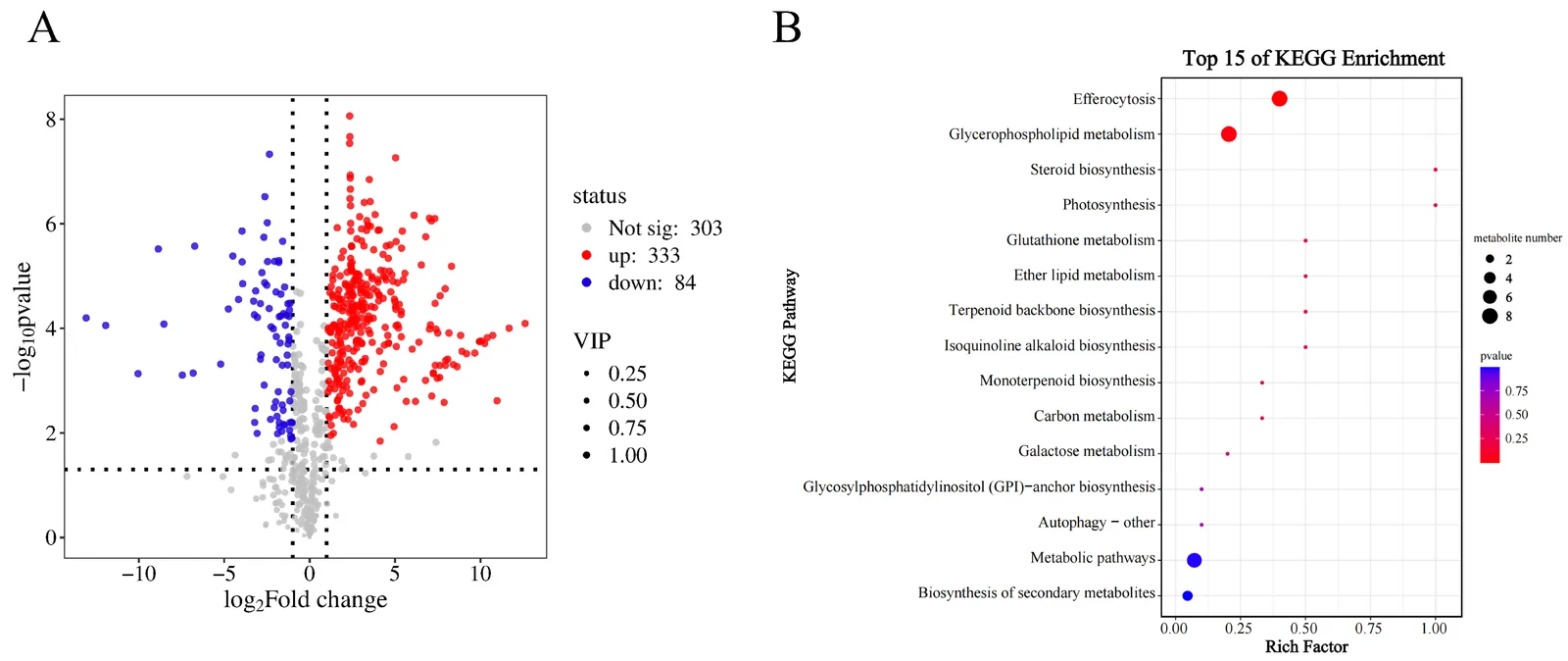
(**A**) Volcano plot of differential metabolites between R-F and R-NF. (**B**) Enriched KEGG pathways based on significantly different metabolites between R-F and R-NF.

In this study, instead of applying False Discovery Rate (FDR) correction, we integrated fold change (FC), *t*-test, and OPLS-DA for a comprehensive multi-perspective analysis of the metabolomic data. Volcano plots were generated using the combined thresholds of *p* < 0.05, FC ≥2 or FC ≤0.5, and VIP ≥1 to illustrate differential metabolites (Figure 4A). The gray dots on the volcano map signified metabolites that did not show significant differences between the R-F and R-NF groups, and the red dots represented metabolites that were significantly up-regulated, while the blue dots represented those that were significantly down-regulated in the R-F group. The results showed that 417 differential metabolites, including 84 downregulated metabolites and 333 upregulated metabolites (Supplementary Table S5). The differential metabolites mainly included the following categories: lipids and lipid-like molecules, amino acids, peptides and analogues, phenylpropanoids and polyketides, heterocyclic compounds, and terpenoids. During fermentation, microorganisms typically degraded and utilized lipid compounds for energy production. The content of 8-(5-hexylfuran-2-yl) octanoylcarnitine (FC = 0.0089), 7-(5-heptylfuran-2-yl) heptanoylcarnitine (FC = 0.0443), and glycerol 1-(9*Z*-octadecenoate) 2-octanoate 3-tetradecanoate (FC = 0.135) decreased significantly as the fermentation progressed. This might be due to microbial growth causing these lipid compounds to be broken down for energy. The reduction in the content of common phospholipids such as PA (P-16:0/20:3(6,8,11)-OH(5)) (FC = 0.1077), PA (18:3(6*Z*, 9*Z*, 12*Z*)/22:6(4*Z*, 7*Z*, 10*Z*, 13*Z*, 16*Z*, 19*Z*)) (FC = 0.1186), and PC (22:6(4*Z*, 7*Z*, 10*Z*, 13*Z*, 16*Z*, 19*Z*)/22:6(5*Z*, 8*E*, 10*Z*, 13*Z*, 15*E*, 19*Z*)-2OH(7*S*, 17*S*)) (FC = 0.2055) may be due to the microbial utilization of these lipids for the synthesis of their own cell membranes during growth and proliferation. Amino acids and oligopeptides could affect the flavor and sensory properties of the fermented product. During the fermentation process, the contents of oligopeptides such as *N*-methoxysuccinyl-Ala-Ala-Pro-Val (FC = 0.0003), Met Leu Gly Gln Thr (FC = 0.292) and Tyr-Gly-Gly-Phe-Gly-OH (FC = 0.362) significantly decreased. The decrease in the content of these oligopeptides may be associated with the reduction in bitterness after fermentation. The increase in the content of oligopeptides, such as Glu-Gln-Leu-Val-Arg (FC = 247.9), Pro-Glu-Arg-Val-Lys (FC = 199.6), Asp-Leu-OH (FC = 5.33), and Pro-Val-Glu-Thr-Leu (FC = 4.66), could enhance the umami and sweet taste, thereby enriching the overall flavor profile and sensory attributes of the fermented paocai. Comparative analysis of phenylpropanoid and polyketide compounds before and after fermentation revealed that most compounds were significantly up-regulated. A total of 35 differential metabolites were identified, including 29 up-regulated and 6 down-regulated compounds. The contents of compounds such as 3-*O*-methylkaempferol (FC = 111.48), kaempferol 3-rungioside (FC = 6.80), catechin 3′,4′-diglucoside (FC = 4.88), and 3-palmitoylcatechin (FC = 3.31) significantly increased after fermentation. This may account for the enhanced antioxidant activity of the fermented paocai.

#### 3.6.4. KEGG Enrichment Analysis

By enriching and analyzing the pathways of differential metabolites, we further screened these pathways to identify the key pathways most closely associated with the differential metabolites. KEGG pathway enrichment analysis revealed significant enrichment in 71 signaling pathways. As shown in Figure 4B, the bubble plot illustrated the top 15 pathways with the highest enrichment, and efferocytosis, glycerophospholipid metabolism, steroid biosynthesis, photosynthesis, glutathione metabolism, ether lipid metabolism, terpenoid backbone biosynthesis and isoquinoline alkaloid biosynthesis were the most enriched KEGG pathways. To elucidate the key pathways underlying fermentation-induced changes in FRR paocai, we focused on glycerophospholipid metabolism, glutathione metabolism, and terpenoid backbone biosynthesis. The glycerophospholipid metabolism pathway was directly involved in regulating the phospholipid composition of cell membranes. During fermentation, microbial utilization of glycerophospholipids may compromise cellular structural integrity, thereby leading to textural softening and reduced crispness of the paocai. The glutathione metabolism pathway was closely associated with the enhanced antioxidant activity observed in FRR paocai after fermentation. FRR was inherently rich in terpenoids, and their contents decreased significantly after fermentation, which may be associated with the reduction in bitterness and astringency in FRR paocai.

### 3.7. Antioxidant Activity

**Figure 5:**
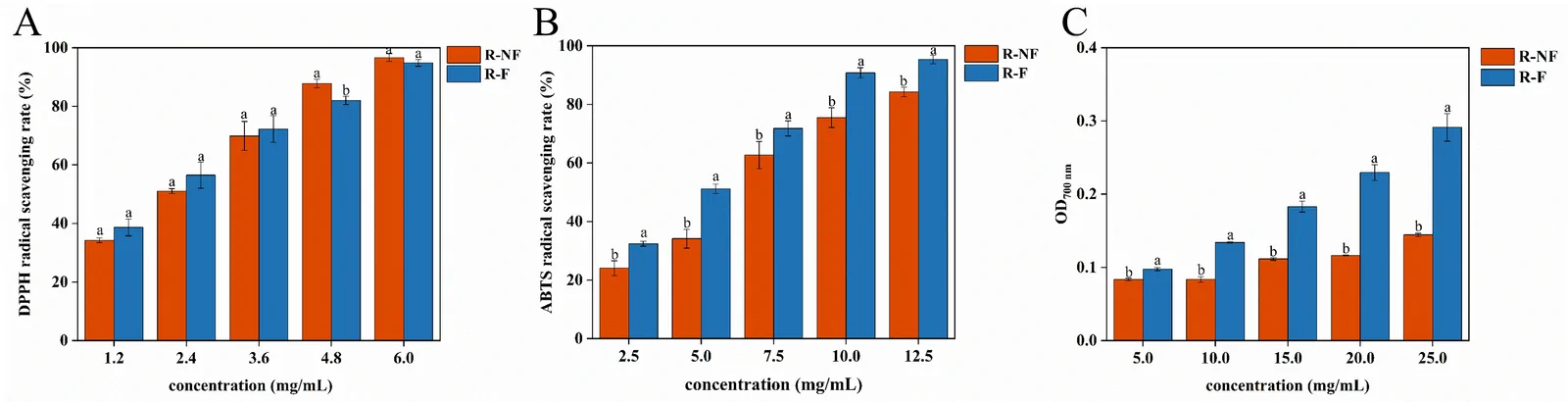
Antioxidant activity of R-F and R-NF. (**A**) DPPH radical scavenging ability. (**B**) ABTS radical scavenging ability. (**C**) FRAP assay. Statistical differences at a 5% significance level were indicated by the superscript letters.

This research utilized three techniques to assess the antioxidant properties of the samples: DPPH, ABTS radical scavenging ability, and FRAP assay. The antioxidant activity of R-F and R-NF is shown in Figure 5. The ability of R-F and R-NF to scavenge DPPH and ABTS radicals improved as their concentrations increased. IC_50_ values help determine the antioxidant effectiveness of a sample, with lower values indicating stronger antioxidant activity [46]. The IC_50_ values for DPPH radical scavenging activity were determined to be 1.794 ± 0.140 mg/mL and 1.963 ± 0.070 mg/mL for R-F and R-NF, respectively. There was no significant difference in DPPH IC_50_ values (*p* > 0.05). According to the study by Yu et al. [47], fermentation did not lead to a significant increase in the DPPH free radical scavenging activity (*p* > 0.05). The IC_50_ values for ABTS radical scavenging activity were determined to be 4.113 ± 0.023 mg/mL for R-F and 5.595 ± 0.142 mg/mL for R-NF. The ABTS IC_50_ differed significantly (*p* < 0.001). The results showed that fermentation significantly enhanced the ABTS radical scavenging ability of FRR (*p* < 0.001), which is consistent with the results of previous studies [48]. As shown in Figure 5C, the absorbance of R-F was significantly higher than that of R-NF, indicating that the antioxidant activity of R-F was superior to that of R-NF (*p* < 0.001). Fermentation enhanced the antioxidant activity of FRR. Overall, FRR paocai exhibited improved antioxidant properties after fermentation. The enhanced antioxidant activity observed after fermentation was likely associated with the increased total phenolic and flavonoid contents.

## 4. Conclusions

FRR has been widely recognized as both a medicinal and edible material due to its remarkable pharmacological activities. However, research on the development of FRR into edible food products remains limited. In this study, we established a multi-dimensional analytical framework integrating response surface methodology, electronic tongue analysis, HPLC, untargeted metabolomics via UPLC-Q-TOF-MS and antioxidant activity assays. This framework was applied to characterize physicochemical changes, flavor development, bioactive compounds, and their corresponding bioactivities of FRR paocai before and after fermentation. We further explored the optimal fermentation process and the impact of fermentation on the quality of FRR paocai. The results showed that the optimal fermentation conditions for FRR paocai were determined to be 5% salt addition, 9% sugar addition, and a fermentation time of 5 days of fermentation at 20 °C. During the fermentation process, the total acidity of FRR paocai gradually increased, while the pH decreased. Fermentation significantly altered the texture of FRR paocai, with crispness, springiness, chewiness, gumminess, and cohesiveness showing a downward trend. The FRR paocai was softer, which was more conducive to chewing. Electronic tongue analyses indicated that fermentation reduced the bitterness and astringency of FRR, while increasing sourness, sweetness, and umami. This eliminated the unfavorable flavor that came with direct consumption of raw FRR and effectively improved the overall sensory quality of the final product. In terms of active ingredients, fermentation treatment significantly improved TPC and TFC and enhanced its antioxidant activity, while reducing the contents of iridoid glycosides and phenylethanoid glycosides in FRR. The reduction in these glycosides may contribute to the decrease in bitterness and astringency;therefore, future work will focus on identifying specific bitter compounds and their sensory impact. Untargeted metabolomics analysis identified a total of 719 metabolites, mainly including lipids, heterocyclic compounds, amino acids and peptides, and terpenoids. Comparison between the R-F and R-NF groups identified 417 differential metabolites, indicating that fermentation significantly altered the metabolic profile of FRR. This study puts forward a sustainable strategy for the value-added utilization of FRR, which is a medicinal and edible resource. Traditionally, FRR processing relies on the cumbersome and time-consuming “nine steaming and nine sun-drying” procedure. In contrast, the fermentation process developed herein offered a simplified and eco-friendly alternative, significantly reducing processing complexity and energy consumption. Furthermore, FRR inherently exhibits poor storage stability, and converting it into paocai effectively extends its shelf life while reducing resource waste caused by spoilage. In conclusion, fermenting FRR into paocai yields a functional food with favorable taste and potent antioxidant activity, which effectively increases the economic value of FRR and promotes the utilization of this valuable biological resource. FRR paocai is produced from a material with both medicinal and edible properties, and offers the following advantages: (1) lactic acid fermentation of FRR effectively reduced the bitterness of raw material, and improved its sensory acceptability for general consumers; (2) during fermentation, microbial enzymes may convert some substances such as phenolics and flavonoids, which enhanced antioxidant activity; (3) for industrial application, low-salt FRR paocai can achieve long-term preservation at room-temperature, reducing raw material waste, extending the deep-processing chain of the characteristic medicinal plants, and forming differentiated health food products with broad market prospects and high industrial conversion potential. Therefore, although this study established a scientific foundation for the development and utilization of FRR resources, further research is still needed, especially regarding the compound probiotic fermentation process and the identification of bitter compounds in FRR.

## Figures and Tables

**Table 1:** Response surface experiment design scheme.

Levels	Factors		
	Salt Addition Amount (A)/%	Sugar Addition Amount (B)/%	Fermentation Time (C)/d
−1	4	8	3
0	5	9	5
1	6	10	7

**Table 2:** The standard curve equations of iridoid glycosides and phenylethanoid glycosides.

Compound	Equation	R^2^ Value
catalpol	*y* = 9.8876*x* + 21.581	0.9999
aucubin	*y* = 19.451*x* + 103.54	0.9993
rehmannioside D	*y* = 9.3555*x* + 24.596	0.9999
melittoside	*y* = 12.269*x* + 45.156	0.9997
ajugol	*y* = 9.9818*x* + 54.062	0.9998
salidroside	*y* = 2.9526*x* + 0.9041	1.0000
acteoside	*y* = 11.539*x* − 16.116	0.9989

**Table 3:** Analysis of the texture changes in paocai during the fermentation process.

	0 d	1 d	3 d	5 d	7 d
crispness (g)	12,632.09 ± 911.21 ^a^	12,433.42 ± 410.25 ^a^	12,104.07 ± 782.53 ^a^	10,894.11 ± 259.73 ^b^	9634.41 ± 253.95 ^c^
springiness (%)	0.58 ± 0.03 ^a^	0.46 ± 0.08 ^b^	0.41 ± 0.08 ^b^	0.41 ± 0.03 ^b^	0.37 ± 0.02 ^b^
chewiness (g)	1925.99 ± 240.01 ^a^	1459.85 ± 522.68 ^ab^	1234.48 ± 330.56 ^bc^	784.84 ± 34.21 ^cd^	476.70 ± 155.39 ^d^
gumminess (g)	3079.26 ± 466.86 ^a^	2935.65 ± 668.64 ^ab^	2590.47 ± 531.83 ^ab^	2044.51 ± 381.72 ^bc^	1290.15 ± 412.71 ^c^
cohesiveness	0.24 ± 0.01 ^a^	0.23 ± 0.07 ^a^	0.25 ± 0.02 ^a^	0.19 ± 0.03 ^ab^	0.13 ± 0.04 ^b^

The values were represented as means ± standard deviation. Statistical differences in the rows at a 5% significance level were indicated by the superscript letters.

**Table 4:** The contents of iridoid glycosides and phenylethanoid glycosides in R-F and R-NF.

Compound	Wavelength	R-NF (mg/g)	R-F (mg/g)
catalpol	203	45.77 ± 0.15 ^a^	0.69 ± 0.08 ^b^
aucubin	203	0.34 ± 0.01 ^a^	0.02 ± 0.00 ^b^
rehmannioside D	203	2.51 ± 0.01 ^a^	0.57 ± 0.01 ^b^
melittoside	203	0.32 ± 0.03 ^a^	0.07 ± 0.01 ^b^
ajugol	203	2.45 ± 0.02 ^a^	0.94 ± 0.00 ^b^
salidroside	275	0.72 ± 0.00 ^a^	0.52 ± 0.01 ^b^
acteoside	334	0.19 ± 0.01 ^a^	0.08 ± 0.00 ^b^

The values were represented as means ± standard deviation. Statistical differences in the rows at a 5% significance level were indicated by the superscript letters.

## Data Availability

The original contributions presented in this study are included in the article/Supplementary Materials. Further inquiries can be directed to the corresponding authors.

## Supplementary Materials

The following supporting information can be downloaded at https://www.mdpi.com/article/10.3390/foods15173097/s1. Figure S1: Total ion chromatogram of R-F and R-NF; Table S1: Sensory evaluation standards; Table S2: Experimental design and results of Box–Behnken response surface method; Table S3: Results of variance analysis; Table S4: All metabolites detected by R-F and R-NF in the negative ion mode; Table S5: Differential metabolites between R-F and R-NF.
