# Supplementary material for: Optimized Paocai Fermentation of Rehmannia Radix: Physicochemical Quality, Untargeted Metabolomics, and Antioxidant Activity
Source: Foods. 2026 Aug 31;15(17):3097. doi: 10.3390/foods15173097 (PMC13565488; doi:10.3390/foods15173097)
Supplement: Supplementary file 1 [file foods-15-03097-s001.zip › Supplementary Figure.pdf]

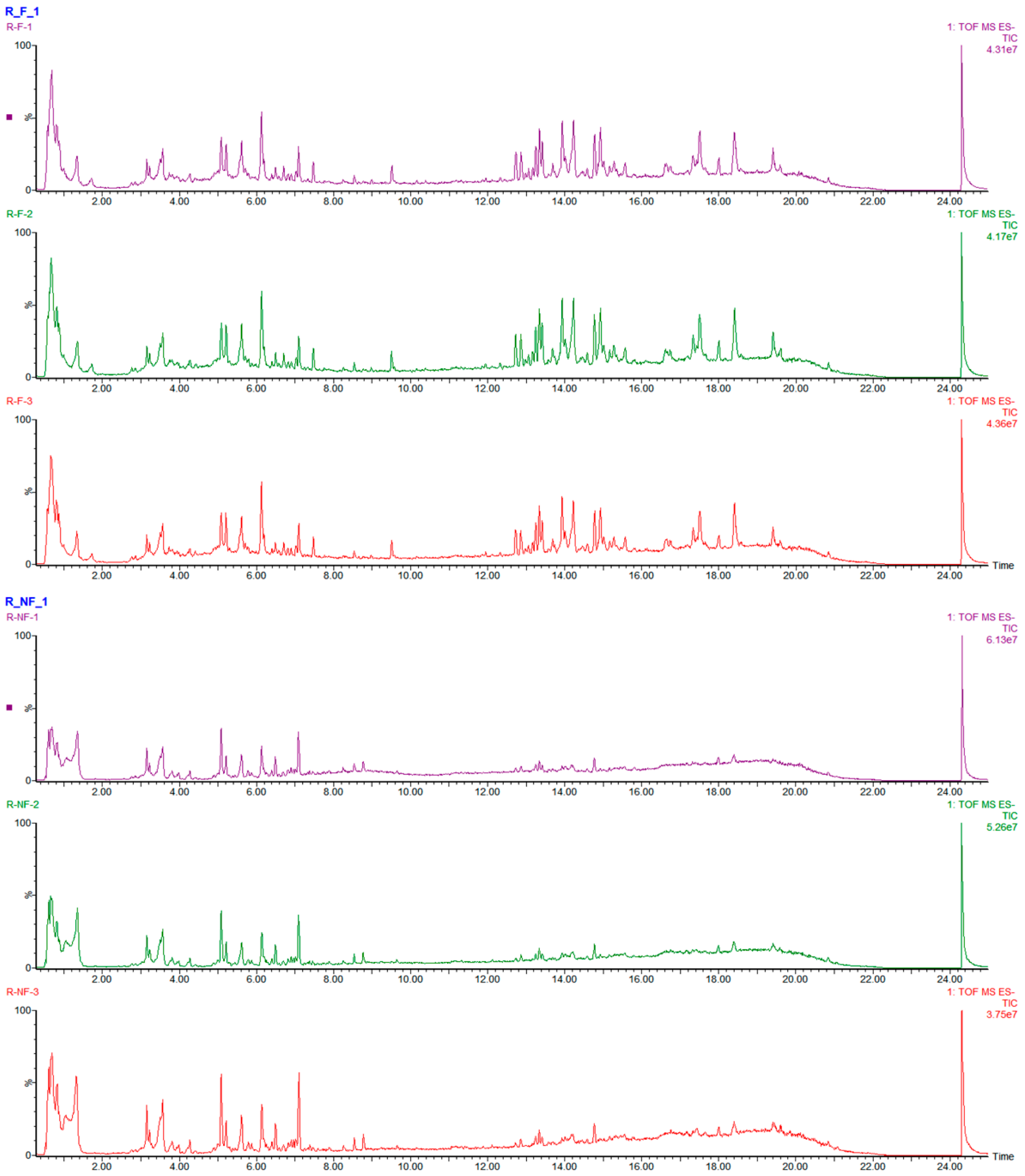

Supplementary Figure S1: Total ion chromatogram of R-F and R-NF
